# Supplementary material for: The Effect of Sex-Specific Differences on IL-10−/− Mouse Colitis Phenotype and Microbiota
Source: Int J Mol Sci. 2023 Jun 20;24(12):10364. doi: 10.3390/ijms241210364 (PMC10299321; doi:10.3390/ijms241210364)
Supplement: Supplementary file 1 [file ijms-24-10364-s001.zip › ijms-2455286-supplementary.pdf]

Title:

Sex-specific difference on IL-$10^{-/-}$ mice colitis phenotype and microbiota

Authors:

Maite Casado-Bedmar, Maryline Roy and Emilie Viennois *

Affiliations:

INSERM, U1149, Center of Research on Inflammation, Université de Paris, Paris, 75018, France

***Correspondence:** emilie.viennois@inserm.fr (E.V.)

ORCID iD:

M.C-B. 0000-0003-0569-6361

E.V. 0000-0002-3104-620X

Supplementary Figure S1. Histological analysis of colonic samples of IL-10$^{-/-}$ mice. Representative images of the histopathological score of H&E stained colonic samples of male (♂) and female (♀) IL-10$^{-/-}$ mice during severe colitis at 17 weeks of age. Scale bar = 250 µm.

Supplementary Figure S2. Taxonomy showing the dysbiosis associated with colitis in IL-10$^{-/-}$ mice. Taxonomic relative abundance at specie level (level 7) for both females and males before colitis (day 0) and during severe colitis (day 91) from fecal samples of IL-10$^{-/-}$ mice.

Supplementary Figure S3. Correlation matrix summarizing all the most relevant variables tested in IL-10$^{-/-}$ mice after 91 days of follow-up, 17 weeks of age. Analysis performed per sex using the Spearman correlation coefficient R, with R = 1 (positive correlation) and R = -1 (negative correlation) displayed with blue or orange, respectively. W/L: weight/length ratio; W/BW: weight/body weight ratio; LPCN-2: fecal lipocalin-2; MPO: colonic myeloperoxidase; TNF: tumor necrosis factor; IL: interleukin; FLGN: flagellin, LPS: lipopolysaccharides; Akkermansia mun.: fecal *Akkermansia muciniphila* at day 91.

Supplementary Figure S1.

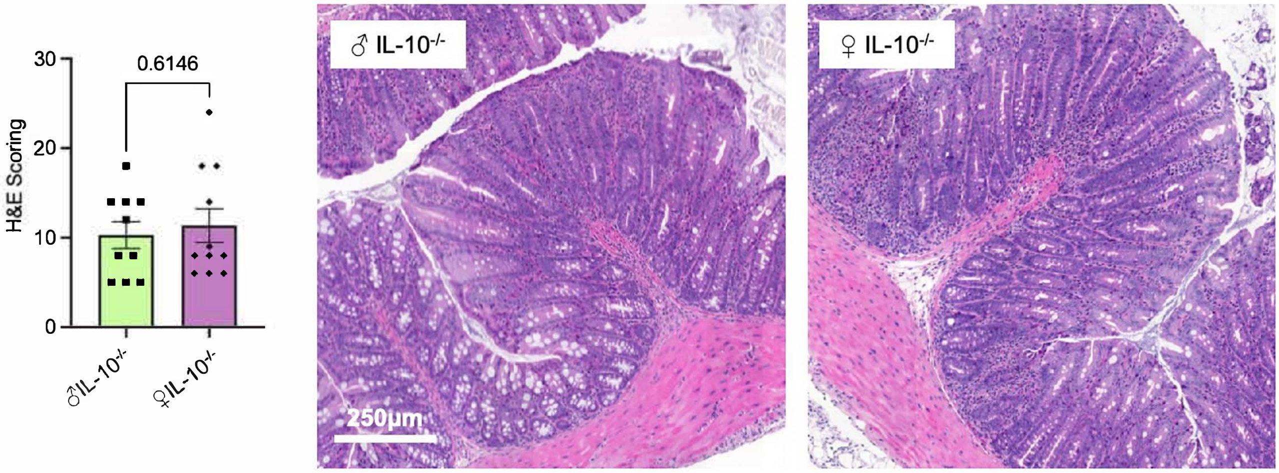

Supplementary Figure S2.

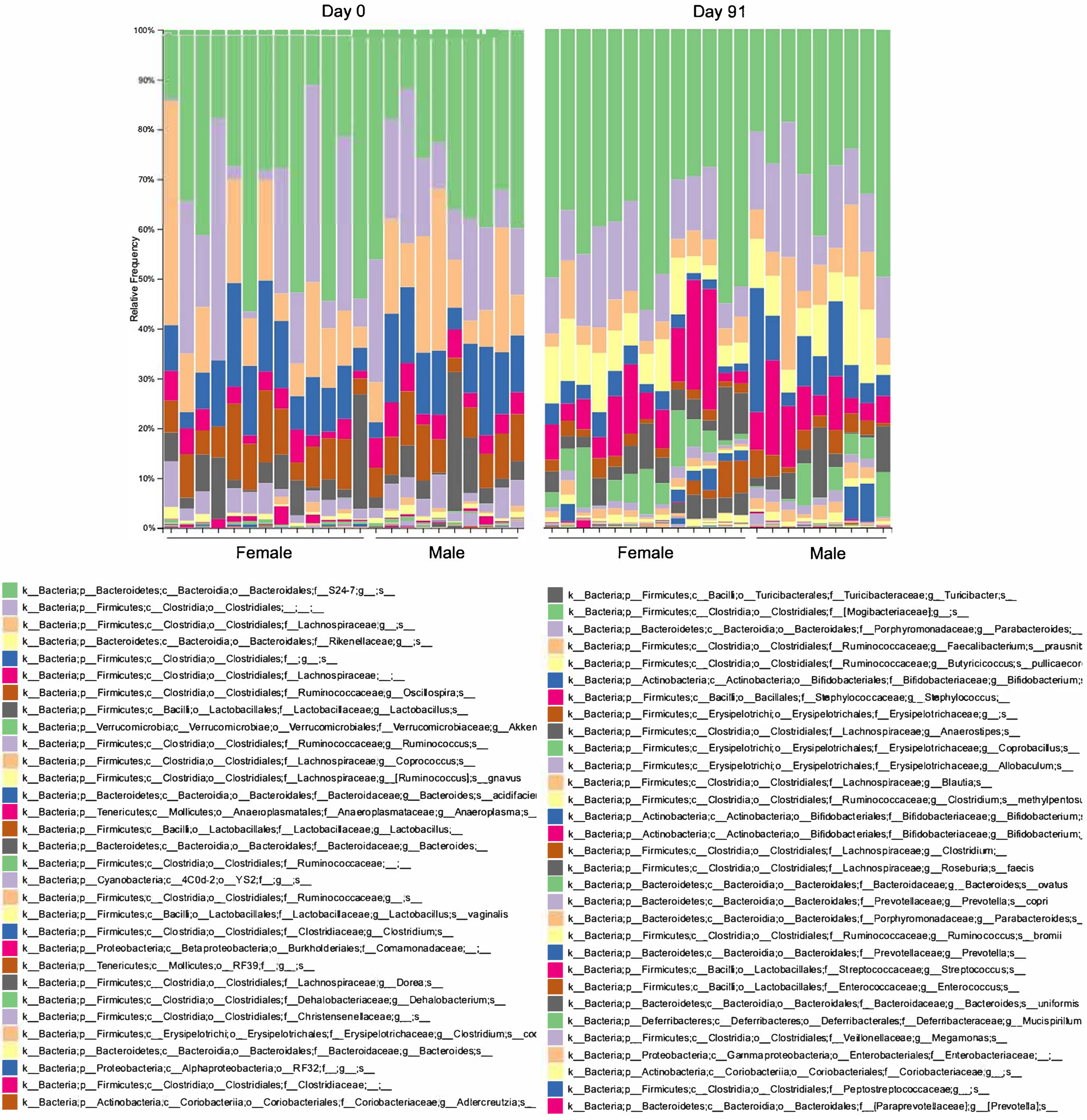

Supplementary Figure S3.

	Colon W/L	Spleen W/BW	LPCN-2	MPO	Colonic miR-21	Fecal miR-21	TNF	IL6	IL1b	FLGN	LPS	Akkermansia mun.
Colon W/L	1.00	0.80	0.41	0.80	0.86	0.46	0.02	0.22	0.38	-0.57	0.03	-0.13
Spleen W/BW	0.80	1.00	0.14	0.83	0.52	0.01	-0.26	0.12	0.05	-0.64	0.08	0.02
LPCN-2	0.41	0.14	1.00	0.31	0.29	-0.15	0.09	0.15	0.48	0.10	0.18	-0.04
MPO	0.80	0.83	0.31	1.00	0.62	0.11	-0.14	0.07	-0.03	-0.54	0.14	0.00
Colonic miR-21	0.86	0.52	0.29	0.62	1.00	0.48	0.13	-0.10	0.21	-0.64	-0.43	-0.15
Fecal miR-21	0.46	0.01	-0.15	0.11	0.48	1.00	0.32	-0.09	0.15	-0.38	-0.34	-0.04
TNF	0.02	-0.26	0.09	-0.14	0.13	0.32	1.00	0.34	0.32	0.05	-0.01	0.07
IL6	0.22	0.12	0.15	0.07	-0.10	-0.09	0.34	1.00	0.71	0.48	0.38	-0.72
IL1b	0.38	0.05	0.48	-0.03	0.21	0.15	0.32	0.71	1.00	0.38	0.12	-0.67
FLGN	-0.57	-0.64	0.10	-0.54	-0.64	-0.38	0.05	0.48	0.38	1.00	0.27	-0.43
LPS	0.03	0.08	0.18	0.14	-0.43	-0.34	-0.01	0.38	0.12	0.27	1.00	0.08
Akkermansia mun.	-0.13	0.02	-0.04	0.00	-0.15	-0.04	0.07	-0.72	-0.67	-0.43	0.08	1.00

Females

	Colon W/L	Spleen W/BW	LPCN-2	MPO	Colonic miR-21	Fecal miR-21	TNF	IL6	IL1b	FLGN	LPS	Akkermansia mun.
Colon W/L	1.00	-0.20	0.47	0.20	0.48	0.12	0.17	-0.80	0.43	0.32	0.22	0.60
Spleen W/BW	-0.20	1.00	-0.02	0.12	-0.15	-0.13	-0.65	0.20	-0.15	0.04	-0.03	-0.25
LPCN-2	0.47	-0.02	1.00	0.37	0.68	0.01	0.45	-0.60	0.45	-0.13	-0.41	0.33
MPO	0.20	0.12	0.37	1.00	0.22	-0.03	-0.08	0.00	0.62	0.15	-0.50	0.12
Colonic miR-21	0.48	-0.15	0.68	0.22	1.00	-0.20	0.37	-0.80	0.27	0.27	-0.21	-0.15
Fecal miR-21	0.12	-0.13	0.01	-0.03	-0.20	1.00	-0.37	0.80	0.44	-0.35	0.28	0.63
TNF	0.17	-0.65	0.45	-0.08	0.37	-0.37	1.00	-0.95	0.10	-0.12	-0.32	0.07
IL6	-0.80	0.20	-0.60	0.00	-0.80	0.80	-0.95	1.00	0.80	-0.40	0.60	1.00
IL1b	0.43	-0.15	0.45	0.62	0.27	0.44	0.10	0.80	1.00	0.15	0.08	0.57
FLGN	0.32	0.04	-0.13	0.15	0.27	-0.35	-0.12	-0.40	0.15	1.00	0.52	-0.12
LPS	0.22	-0.03	-0.41	-0.50	-0.21	0.28	-0.32	0.60	0.08	0.52	1.00	0.38
Akkermansia mun.	0.60	-0.25	0.33	0.12	-0.15	0.63	0.07	1.00	0.57	-0.12	0.38	1.00

Males

1.0 / 0.5 / 0 / -0.5 / -1.0
